# Regulation of *Myf5* Early Enhancer by Histone Acetyltransferase p300 during Stem Cell Differentiation

**DOI:** 10.4172/2168-9547.1000103

**Published:** 2012-03-17

**Authors:** Tanja Francetic, Melanie Le May, Munerah Hamed, Hymn Mach, David Meyers, Philip A Cole, Jihong Chen, Qiao Li

**Affiliations:** 1 Departments of Pathology and Laboratory Medicine, University of Ottawa, Ottawa, ON Canada; 2 Cellular and Molecular, Medicine, Faculty of Medicine, University of Ottawa, Ottawa, ON Canada; 3 Department of Pharmacology and Molecular Sciences, Johns Hopkins University School of Medicine, Baltimore, MD USA

**Keywords:** Gene regulation, Transcription factor, Acetyltransferase, Histone acetylation, Stem cell differentiation, Regulatory elements

## Abstract

Skeletal myogenesis is an intricate process coordinated temporally by multiple myogenic regulatory factors (MRF) including Myf5, which is the first MRF expressed and marks the commitment of skeletal muscle lineage. The expression of Myf5 gene during early embryogenesis is controlled by a set of enhancer elements, and requires the histone acetyltransferase (HAT) activity of transcriptional coactivator p300. However, it is unclear as to how different regulatory signals converge at enhancer elements to regulate early Myf5 gene expression, and if p300 is directly involved. We show here that p300 associates with the *Myf5* early enhancer at the early stage of stem cell differentiation, and its HAT activity is important for the recruitment of β-catenin to this early enhancer. In addition, histone H3-K27 acetylation, but not H3-K9/14, is intimately connected to the p300 HAT activity. Thus, p300 is directly involved in the regulation of the *Myf5* early enhancer, and is important for specific histone acetylation and transcription factor recruitment. This connection of p300 HAT activity with H3-K27 acetylation and β-catenin signalling during myogenic differentiation *in vitro* offers a molecular insight into the enhancer-elements participation observed in embryonic development. In addition, pluripotent stem cell differentiation is a valuable system to dissect the signal-dependent regulation of specific enhancer element during cell fate determinations.

## Introduction

Skeletal myogenesis *in vivo* is coordinated temporally by multiple myogenic regulatory factors (MRF), among which Myf5 is the first to be expressed and marks the commitment of skeletal muscle lineage [1,2]. The expression of Myf5 gene during embryogenesis is controlled by a set of enhancer elements [3–5]. The genomic location and multitude of *Myf5* enhancers are well characterized. However, less is known as to how different regulatory signals and transcription factors converge at enhancer elements to regulate Myf5 gene expression during different phases of skeletal myogenesis [2].

The transcriptional coactivator p300 has an intrinsic histone acetyltransferase (HAT) activity, and is essential for a myriad of cellular processes, including myogenesis [6,7]. The HAT activity of p300 is important for heart, lung, and small intestine development in mouse embryos [8]. In addition, p300 is indispensable for the expression of MRF genes, and is therefore critically required for skeletal myogenesis *in vivo*. Knock-out of p300 abrogates MRF gene expression and skeletal muscle formation in mouse and ES cell systems [9]. More importantly, knock-in of a p300 HAT mutant is sufficient to attenuate the gene expression of Myf5 and MyoD, and to severely impair skeletal myogenesis [9]. Nonetheless, the HAT activity of CBP, a functional homologue of p300, is not essential for this process [9]. Interestingly, the expression of Pax3 which acts at the upstream of MRFs, is not affected by a loss of p300 HAT activity [9]. Thus genetic evidence in mouse and ES cell models put p300 hierarchically upstream of Myf5 and MyoD by establishing that Myf5 and MyoD gene expression specifically require p300 HAT activity [9]. However, on a molecular level, whether p300 HAT activity is directly involved in the regulation of Myf5 and MyoD gene expression remains to be determined.

Canonical Wnt/β-catenin signalling is also important for embryonic development and skeletal myogenesis. Wnt signalling regulates *Myf5* gene expression at the onset of myogenesis, and Myf5 is a direct target of β-catenin [10,11]. While Wnt controls the stability and cellular localization of β-catenin, Tcf/Lef transcription factors recruit β-catenin to the target genes for transcriptional activation [12]. Furthermore, p300 acts as a coactivator of β-catenin. In that, it interacts with β-catenin, acetylates β-catenin, and synergistically activates β-catenin/Tcf-mediated transformation [13–15]. The expression of Myf5 in the epaxial dermomyotome is regulated by an early epaxial enhancer [16,17], and several Lef/Tcf sequences flanking the early enhancer determine the correct spatiotemporal expression of Myf5 in the epaxial somite [11]. The Myf5 early epaxial enhancer is also known to be regulated by Dmrt2 [18].

Pluripotent P19 embryonal carcinoma (EC) cells, like embryonic stem (ES) cells, respond well to development cues *in vitro*, to differentiate into cell types of all three germ layers [19]. Specifically, the commitment of P19 EC cells into skeletal muscle lineage recapitulates closely the cellular and molecular processes occurring in early embryogenesis and ES cell differentiation [20–23]. Pax3, a progenitor factor, is expressed at the early stage of differentiation, while MyoD and myogenin are subsequently expressed marking the development of skeletal myocytes [22,23]. This resemblance of P19 differentiation with skeletal myogenesis *in vivo*, and their ease differentiation in large scale have made them a valuable differentiation system to study the mechanisms of lineage specification [23,24].

In this study, we have examined the requirement of p300 HAT activity for early Myf5 gene expression with respect to β-catenin occupancy and histone acetylation at the *Myf5* enhancer during the early stage of P19 cell differentiation. Our studies have demonstrated that p300 is directly involved in the early regulation of *Myf5* enhancer. Furthermore, the p300 HAT activity is intimately connected with specific histone acetylation and β-catenin signalling in the regulation of *Myf5* early enhancer.

## Results

### Histone acetylation during myogenic specification

**Figure 1:**
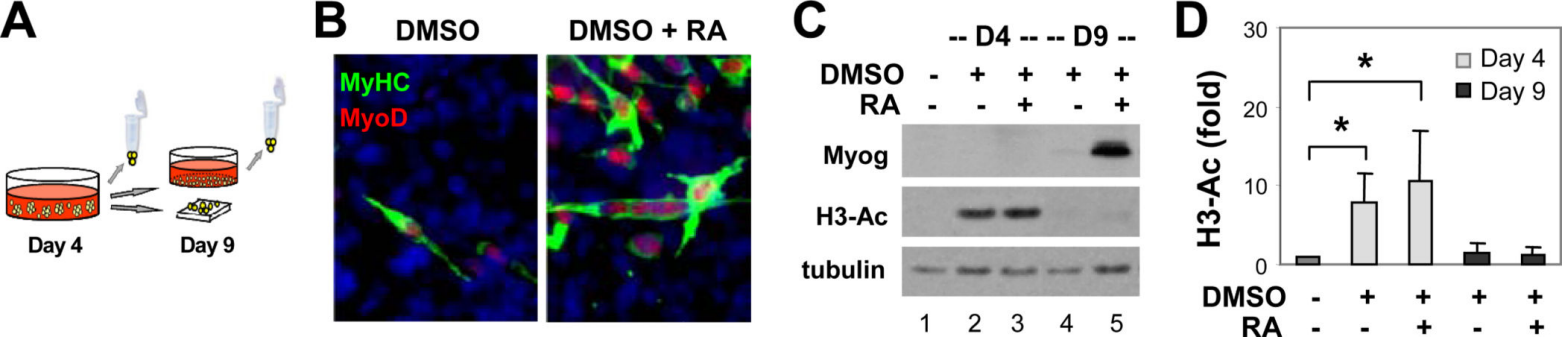
Histone acetylation and myogenic differentiation. (A) Schematic presentation of the aggregation protocol for P19 cell differentiation. Cells were treated with DMSO in the presence or absence of RA (10 nM) during EB formation and maintained as adhesive culture for additional 5 days without treatments to develop skeletal myocytes. (B) The cells were stained on day 9 with specific antibodies for microscopic analysis of MyoD (red), myosin heavy chain (MyHC, green), and with Hoechst to visualize nuclei (blue). (C) Myogenin gene expression and global histone H3 acetylation (H3-Ac) on day 4 and day 9 of differentiation was examined by Western blot analysis. (D) Quantification of the H3 acetylation blots is presented as fold changes in relation to the undifferentiated control (mean ± SD, n = 3). Statistical significance is denoted by * to indicate p < 0.05 relative to the undifferentiated control.

P19 pluripotent stem cells have been used extensively to study the molecular mechanisms of cellular differentiation [22–24]. In tissue cultures, P19 cells can be induced into myogenic differentiation with an aggregation protocol (Figure 1A) which involves the formation of embryonic bodies (EBs) and the use of small molecule inducers [23,24]. As previously reported, treatment with DMSO during EB formation induced the commitment of P19 cells into skeletal myocytes in a relatively low efficacy, and the elongated bipolar skeletal myocytes developed by day 9 of differentiation (Figure 1B). Cotreatment of the EBs with all-*trans* retinoic acid (RA) significantly enhanced the development of skeletal myocytes, which also exhibited a more intensive staining of myosin heavy chain as revealed by the immune fluorescence microscopic analysis (Figure 1B). In addition, MyoD protein co-stained with myosin heavy chain in the developing myocytes (Figure 1B) and the myogenin protein, an identity marker of skeletal myocytes, was detected by Western blot analysis by day 9 (Figure 1C).

Intriguingly, we also detected a significant increase in the level of global histone H3 acetylation on day 4 of differentiation following DMSO induction compared to the undifferentiated cells (Figure 1C and D). The addition of RA further increased the level of H3 acetylation, but it scaled back to a similar level as in undifferentiated cells by day 9 of differentiation (Figure 1C and D). Thus, RA enhances skeletal myogenesis possibly through signalling histone acetylation at the early stage of P19 stem cell differentiation, the stage of lineage specification or EB formation.

### Curcumin inhibits myogenic specification

**Figure 2:**
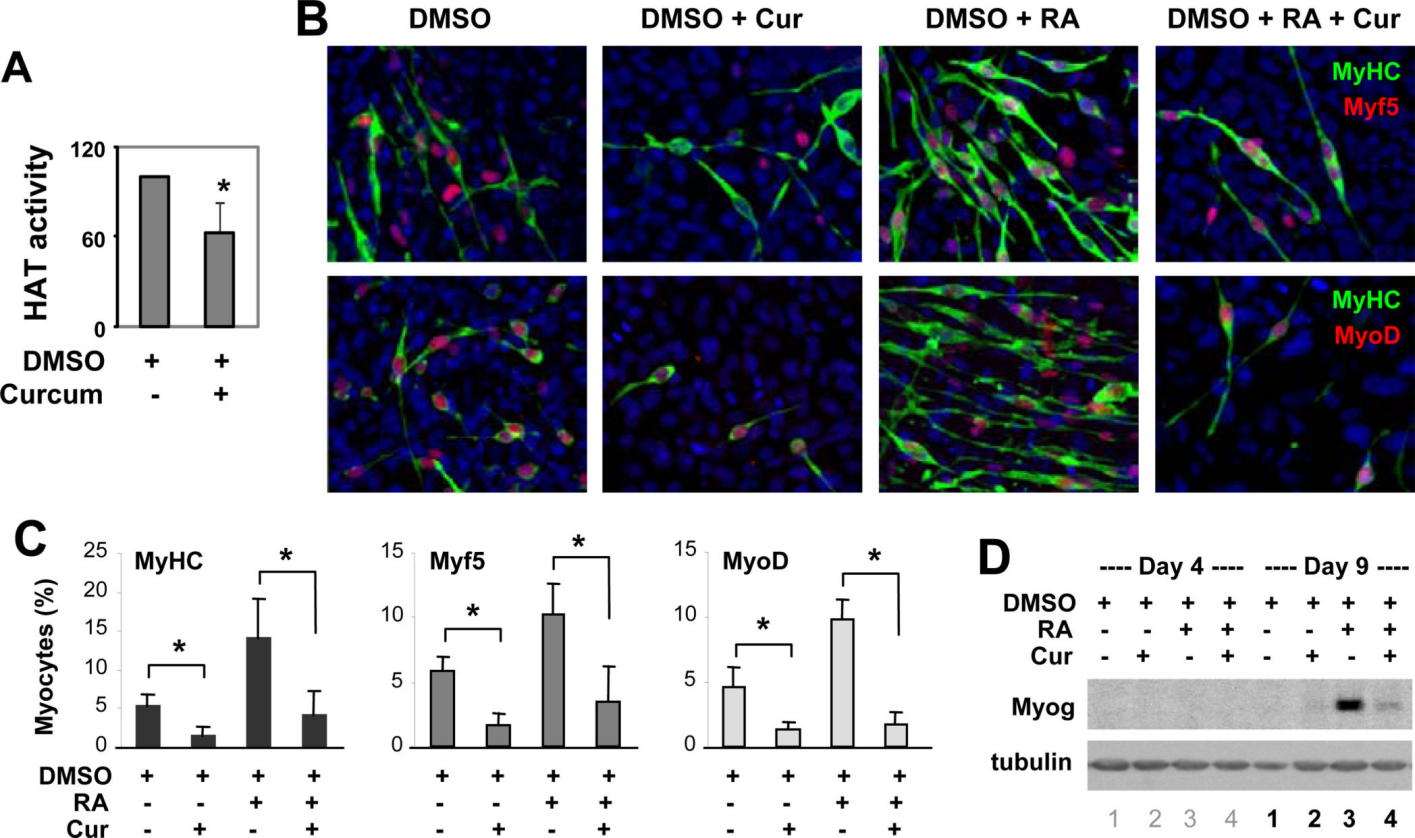
Curcumin inhibits myogenic differentiation. (A) Cells were treated with curcumin (Cur, 10 µM) during EB formation. Endogenous p300 was immunoprecipitated on day 4 and its HAT activity was examined by HAT assay. Quantification of the p300 HAT activity was presented as percentage of the DMSO control. Error bars represent the standard deviation (n = 5, * P < 0.05). (B) Cells were differentiated with DMSO or RA (10 nM) in the presence or absence of curcumin during EB formation and co-stained for myosin heavy chain (MyHC) and Myf5 on day 9, and with Hoechst to visualize nuclei (blue). The cells were also co-stained for myosin heavy chain and MyoD in parallel. (C) Quantifications of myocytes are presented as the fractions of cells stained positively for myosin heavy chain, Myf5 or MyoD (mean ± SD, n = 3) in relation to the total cell populations. Statistical significance is denoted by * to indicate p < 0.05. (D) The expression of myogenin protein on day 4 and day 9 of differentiation was determined by Western blot analysis.

To delineate the interplay of histone acetylation and nuclear HAT activity during myogenic differentiation, we used curcumin, which inhibits the HAT activity of p300 and CBP, but not PCAF [25]. We tested different concentrations of curcumin (1–100 µM) in P19 stem cells, and administered 10 µM in our studies, since it did not exhibit apparent toxicity to the differentiating cells, while still inhibiting about 40% of p300 HAT activity as determined by the HAT assay (Figure 2A). Next, we examined the effects of curcumin on myogenic differentiation, particularly at the stage of lineage specification.

Cells were grown in suspension and treated with curcumin in the presence of DMSO and RA during EB formation, maintained as adhesive cultures without any treatments for additional 5 days, and then analyzed for the efficacies of myogenic differentiation. As shown in figure 2B and C, curcumin administration significantly decreased both the DMSO-induced and RA-enhanced myogenic differentiation by about 70%, as determined by quantitative analysis of myosin heavy chain positive cells. We also co-stained the cells for Myf5 or MyoD protein in parallel. Following curcumin administration, the fractions of cells expressing Myf5 and MyoD decreased by 65 and 80% respectively (Figure 2B and 2C). In addition, the expression of myogenin protein, an identity marker of skeletal myocytes, was also severely impaired by the curcumin treatment (Figure 2D). Thus, treatment with curcumin at the early stage of differentiation, or during EB formation, is sufficient to inhibit the commitment of stem cells into muscle lineage. This negative effect of curcumin on myogenic differentiation may stem from its property as a HAT inhibitor, since p300 HAT activity is required for gene expression of Myf5 and MyoD in mouse model systems [9].

### C646 also inhibits myogenic specification

**Figure 3:**
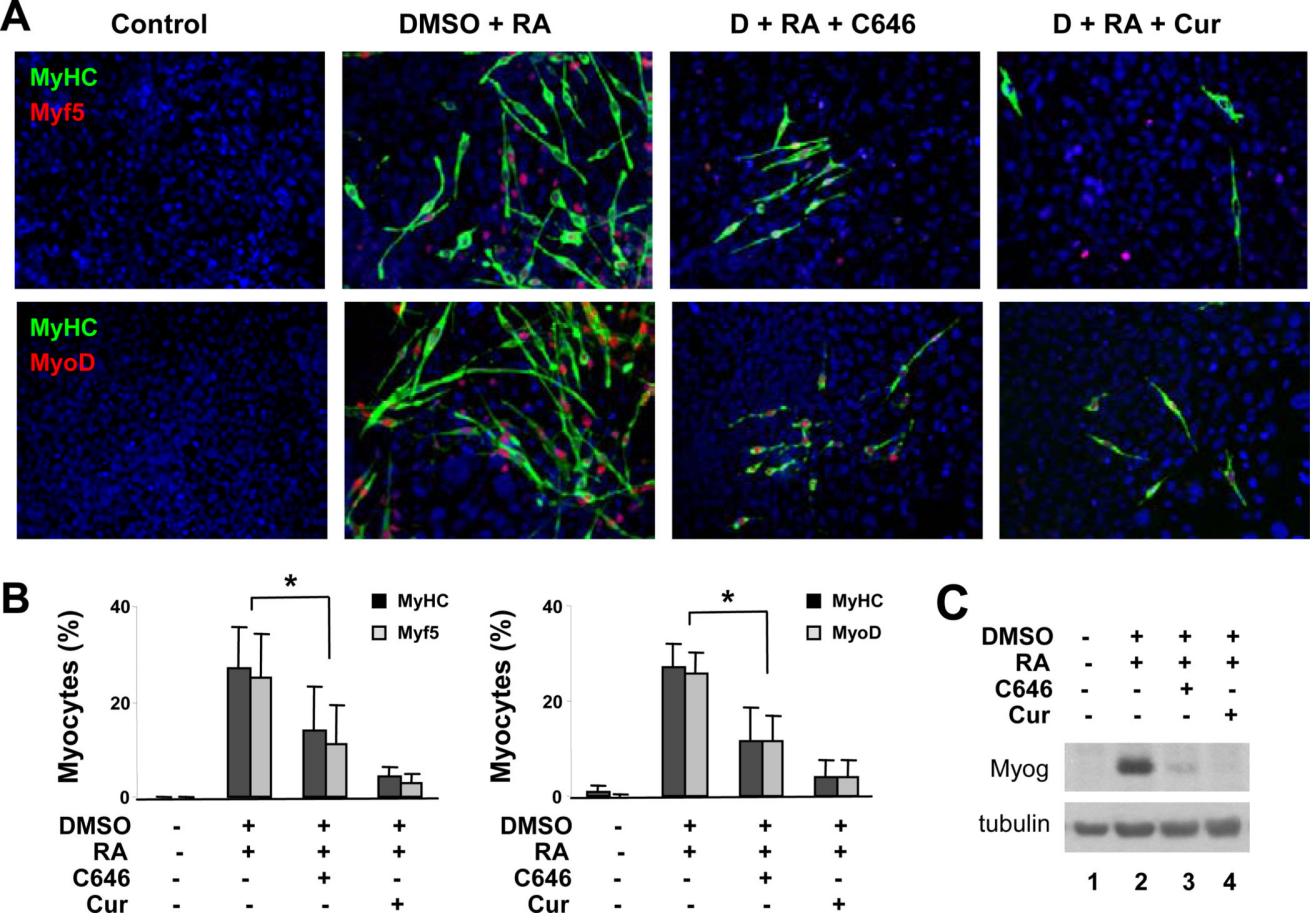
C646 inhibits myogenic differentiation. Cells were differentiated with DMSO and RA (10 nM) in the presence or absence of C646 (10 µM) during EB formation and cultured for an additional 5 days without any treatment. Curcumin (Cur, 10 µM) was used in parallel as comparison. The cells were co-stained for myosin heavy chain (MyHC) and Myf5, or myosin heavy chain and MyoD on day 9, and with Hoechst to visualize nuclei (blue). (B) Quantifications are presented as fractions of cells positively stained for myosin heavy chain, Myf5 or MyoD (mean ± SD, n = 4). (C) Myogenin expression was determined by Western blot analysis.

To further determine the contribution of nuclear HAT activity to myogenic differentiation, we also employed a newly identified p300 HAT inhibitor, C646, which is a competitive inhibitor highly selective to p300 [26]. Cells were treated with C646 (2–20 µM) in the presence of DMSO and RA during EB formation, and then analyzed for the efficacies of myogenic differentiation. Curcumin was also administered in parallel as comparison. As shown in figure 3A and 3B, C646 treatment (10 µM) significantly decreased RA-enhanced myogenic differentiation, to a comparable level obtained with the curcumin treatment as determined by quantitative microscopic analysis of myosin heavy chain positive cells. Likewise, the fractions of cells expressing Myf5 or MyoD protein were also significantly decreased following C646 treatment (Figure 3A and B). In addition, C646 treatment impaired the expression of myogenin protein as shown by the Western blot analysis (Figure 3C). Thus C646, similar to curcumin, inhibits myogenic differentiation at the stage of lineage specification. Again, this negative effect may be a result of its property as an inhibitor of p300 HAT activity which is necessary for the gene expression of MyoD and Myf5 in mouse model systems [9].

### Curcumin and C646 attenuate Myf5 transcripts

**Figure 4:**
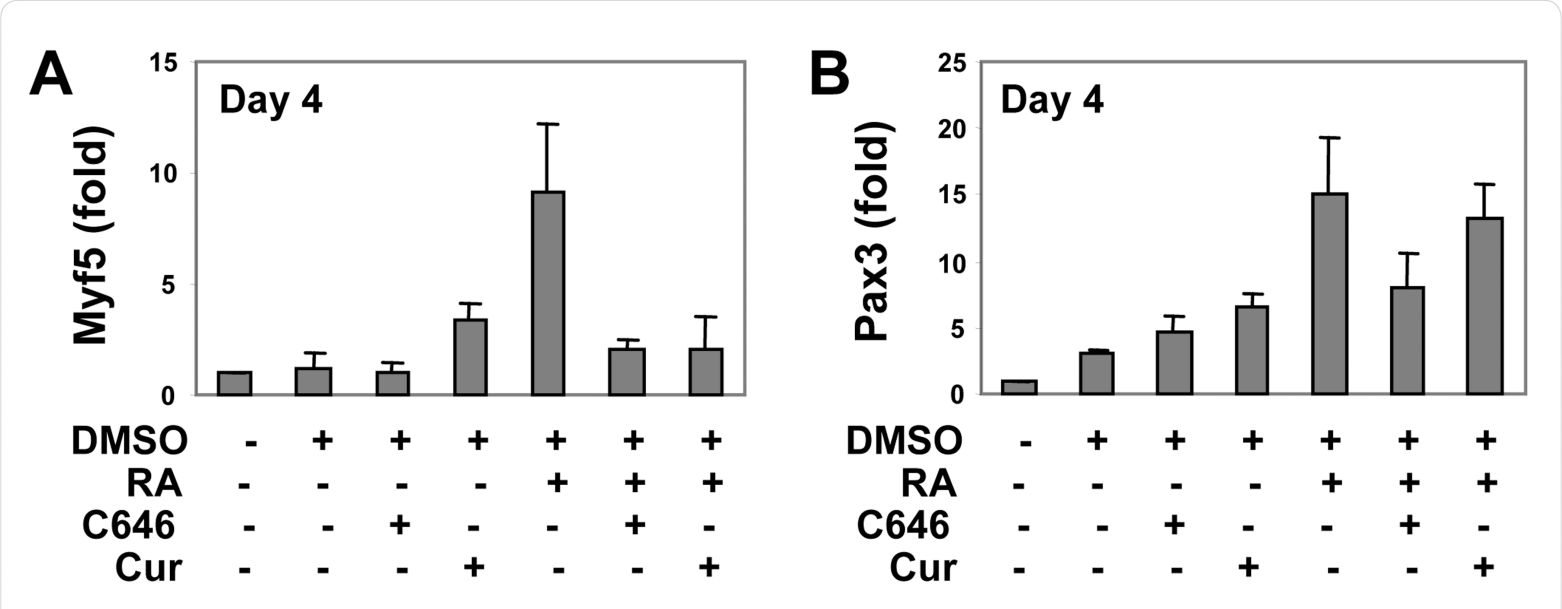
Curcumin and C646 decrease the transcripts of Myf5 but not Pax3. (A) Cells were differentiated with RA (10 nM) in the presence of C646 (10 µM), or curcumin (Cur, 10 µM) during EB formation. The relative mRNA levels of Myf5 on day4 of differentiation were determined by quantitative real-time RTPCR, and plotted as the fold changes in relation to the untreated controls after being normalized to GAPDH (mean ± SD, n = 3). (B) The relative mRNA levels of Pax3 on day 4 of differentiation were also determined by quantitative realtime RT-PCR and plotted as the fold change relative to the untreated control after being normalized to GAPDH (mean ± SD, n = 3).

During P19 myogenic differentiation, the transcript level of progenitor factor Pax3 increases by day 4 of differentiation following RA administration [22,23]. Myf5 is the first MRF to be expressed, which marks the commitment of progenitor cells into skeletal muscle lineage [2]. We found that similar to Pax3, Myf5 transcripts were also detected by day 4 of differentiation (Figure 4A). More importantly the addition of RA significantly increased the level of Myf5 transcripts as determined by quantitative real time RT-PCR analysis (Figure 4A).

The inhibition of myogenic differentiation by curcumin and C646 were attained with treatments at the early stage of myogenic differentiation, prior to the expression of MyoD [23], suggesting that Myf5 gene expression, which is at the upstream of MyoD, may be negatively affected. We therefore examined the impact of curcumin and C646 on Myf5 gene transcripts. As shown in figure 4A, the addition of RA increased the level of Myf5 transcripts by about 9-fold, in comparison to the untreated control. Co-treatment with C646 or curcumin effectively attenuated the increase of Myf5 transcripts by RA (Figure 4A). Thus C646 and curcumin inhibit the commitment of skeletal muscle lineage at the level of Myf5 gene transcription.

Interestingly, while RA also increased the level of Pax3 transcripts by day 4 of differentiation, cotreatment with curcumin or C646 has lesser impact on the increase of Pax3 transcripts by RA (Figure 4B). Taken together, our data are in agreement with mouse model studies in which p300 HAT activity is essential for Myf5 and MyoD gene expression, but not for the progenitor factor Pax3 [9]. Since C646 and curcumin are inhibitors of p300 HAT activity, it is possible that the decrease in Myf5 gene transcripts is due to their negative effects on the HAT activity of p300.

### Curcumin decreases p300 and β-catenin occupancy at the *Myf5* early enhancer

Although the HAT activity of p300 is essential for Myf5 gene expression in embryonic development, it is not known if p300 is directly involved in the regulation of *Myf5* enhancer [9]. The spatiotemporal activation of Myf5 *in vivo* is controlled by a set of enhancers [2] and the early expression of Myf5 in mouse is regulated by an epaxial enhancer [16,17]. Since curcumin and C646 treatments during EB formation were sufficient to inhibit Myf5 gene expression (Figure 2–4), we next assessed the occupancy of p300 at this early enhancer by a real-time PCR based chromatin immunoprecipitation (ChIP) assay. Our goal was to determine whether p300 is directly participates in the regulation of *Myf5* enhancer elements at the early stage of myogenic differentiation.

**Figure 5:**
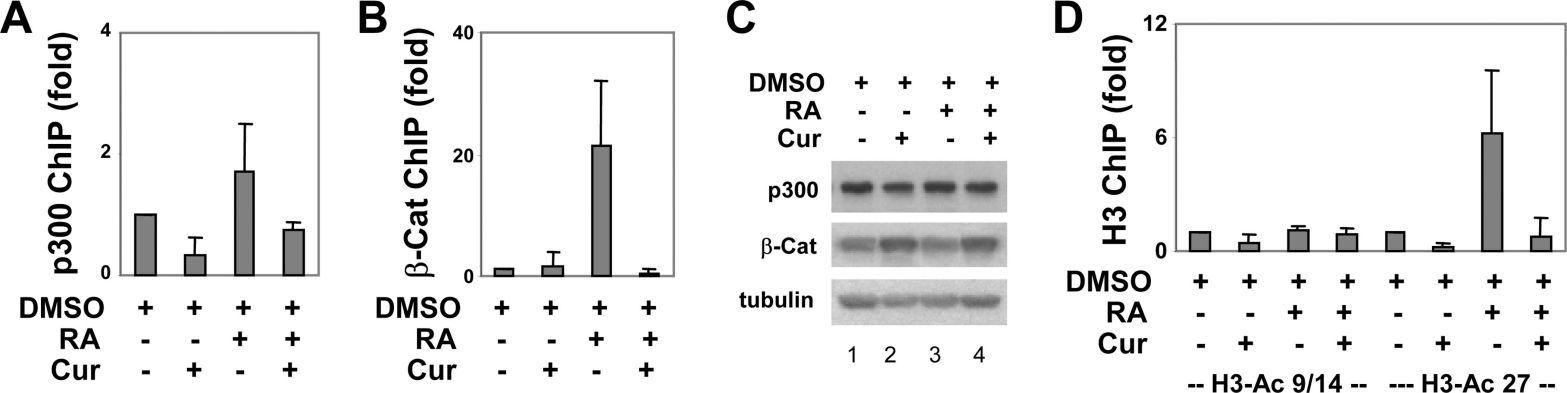
Interplay of p300 and β-catenin occupancy with histone acetylation at the *Myf5* early enhancer. (A) Cells were grown as EBs and induced to differentiate with DMSO and RA (10 nM) in the presence or absence of curcumin (10 µM). The occupancy of the *Myf5* early enhancer by p300 was determined by a real-time PCR based ChIP assay on day 4 and presented as the fold changes in relation to the DMSO control. Input DNA was used as internal controls (mean ± SD, n = 3). (B) The association of β-catenin (β-Cat) with this enhancer was also analyzed and presented as in panel A (mean ± SD, n = 4). (C) The levels of p300 and β-catenin protein on day 4 of differentiation were examined by Western blot analysis. β-tubulin was used as a loading control. (D) The levels of histone acetylation on lysine 9/14 (H3-AC 9/14) and 27 (H3-Ac 27) at the *Myf5* early enhancer were determined by the ChIP assay and plotted as the fold changes in relation to the DMSO controls (mean ± SD, n = 3).

As shown in figure 5A, p300 was detected at the *Myf5* early enhancer in EBs treated with DMSO alone or in combination with RA. The occupancy of p300 increased about 2-fold in EBs treated with additional RA compared with the DMSO alone control (Figure 5A). Co-treatment with curcumin decreased p300 occupancy by about 60% at this early enhancer in EBs treated with DMSO alone or with additional RA, but it is not statistically significant (Figure 5A). Thus, p300 is directly involved in the regulation of the *Myf5* early enhancer, possibly acting as a hallmark of this enhancer.

The *Myf5* early epaxial enhancer is also regulated by β-catenin signalling pathway, and p300 is a coactivator of β-catenin [11,13,15]. In addition, the function of β-catenin is necessary for RA-enhanced skeletal myogenesis [22,23]. However, little is known about the interplay of β-catenin and RA signalling at the level of enhancer regulation, particularly during stem cell differentiation. We next examined the impact of curcumin on the association of β-catenin with the *Myf5* early enhancer by the ChIP assay. As shown in figure 5B, β-catenin associated with this enhancer, which increased markedly, by about 20-fold, after RA addition. Intriguingly, curcumin treatment abrogated this RA enhanced β-catenin association (Figure 5B). More importantly, the abrogation of β-catenin association with the early enhancer by curcumin was not due to a lack of β-catenin protein, since the levels of cellular β-catenin protein remained constant regardless of treatments (Figure 5C). Thus, the inhibitory effects of curcumin on skeletal myogenesis may also be mediated by a deactivation of β-catenin transcriptional activity, in connection to an inhibition of p300 HAT activity.

### Specific histone acetylation at the *Myf5* early enhancer

Since p300 HAT activity is necessary for Myf5 gene expression [9] and one basic function of p300 on chromatin is to acetylate histones [7], we also examined the status of histone acetylation at the *Myf5* early enhancer by the ChIP assay, particularly during the early stage of stem cell differentiation. As shown in figure 5D, the levels of acetylated histone H3 at lysine 9/14 (H3-K9/14) at the *Myf5* early enhancer remained constant regardless of treatments. However, a marked increase in H3-K27 acetylation, about 6-fold, was detected following RA administration (Figure 5D). More importantly, curcumin treatment effectively impeded the acetylation of H3-K27 at this enhancer (Figure 5D). Thus the function of p300 at the *Myf5* early enhancer is to acetylate histone at specific lysine residues, and the negative effects of curcumin on myogenic differentiation may be a result of its inhibitory effect on the p300 HAT activity. Taken together, our results suggest that the HAT activity of p300 is directly involved in specific histone acetylation to regulate the *Myf5* early enhancer during pluripotent stem cell differentiation.

## Discussion

In this study, we show that the regulation of early expression of Myf5 gene during stem cell differentiation recapitulates the mechanisms of enhancer-element participation observed *in vivo*. The transcriptional coactivator p300 associates with the *Myf5* early enhancer at the early stage of stem cell differentiation, and its HAT activity is essential for the recruitment of β-catenin to this early enhancer. In addition, the acetylation of H3-K27, but not H3-K9/14, is intimately connected with the p300 HAT activity and the β-catenin recruitment at this early enhancer. Thus, the HAT activity of p300 is directly involved in the early regulation of *Myf5* enhancer, and is critical for both specific histone acetylation and transcription factor recruitment at the *Myf5* early enhancer.

In eukaryotic cells, *cis*-regulatory elements, such as insulators, enhancers and promoters, are organized with histones, and further packaged into a higher order of chromatin structure [27–30]. The organization of chromatin establishes hierarchical platforms on both local and global levels for intricate regulatory-protein interactions during cell fate determinations, and ultimately for the control of transcription programs. Recent genome-wide analyses have demonstrated an apparent functional relationship between chromatin dynamics and transcriptional activities during lineage specification. For instance, enhancers are generally associated with p300 and histone acetylation [31,32]. Epigenetic characteristics or chromatin signatures, therefore, are marks of activated regulatory elements [33–35].

Transcriptional coactivator p300 is important for skeletal myogenesis, but its functional modes differ in different stages of skeletal myogenesis. For example, following the expression of early MRFs, p300 acetylates MyoD and functions as a coactivator of MyoD, which is necessary to activate the down-stream myogenic program, including myogenin gene expression, and to facilitate skeletal muscle development [36–39]. In addition, p300 is also involved in the early stage of myogenic differentiation, the specification of muscle lineage, since p300 HAT activity is indispensable for Myf5 and MyoD gene expression in mouse models [9].

We show in this study that p300 in fact is directly involved in the early regulation of the *Myf5* enhancer at the stage of lineage specification, particularly at the early enhancer (Figure 5). We have previously studied the recruitment of p300 to a *Pax3* locus by retinoic acid receptor (RAR) during RA-enhanced myogenic differentiation [23]. RA enhances Pax3 gene expression, while increasing significantly (about 15-fold) the recruitment of p300 to this locus, which contains a RA responsive element for direct RAR-binding. Interestingly, the association of p300 with the *Myf5* early enhancer, which appears not to be a direct target of RAR binding, increases moderately (about 2-fold) following RA treatment (Figure 5A). These data suggest an indirect mechanism of RA signalling, in which p300 acts as a hallmark at the *Myf5* early enhancer. Although the association of p300 with the *Myf5* early enhancer may not be mediated directly by DNA bound RAR, or may not be under a direct mechanism of RA signalling (Figure 5), p300 HAT activity seems to be regulated by RA.

The spatial-temporal expression of Myf5 gene during embryogenesis is a complex process, and a large number of enhancer elements ensure Myf5 expression at specific locations under right timing in the developing embryo [2]. While, *in vivo* studies have shown where and when Myf5 is expressed, little is known about the molecule mechanisms by which each individual enhancer is regulated. We show that inhibition of p300 HAT activity in the P19 differentiation system closely recapitulates the negative effects of inactivating p300 HAT activity on skeletal myogenesis of mouse models (Figure 2–4). In addition, the *Myf5* early enhancer is directly affected by p300 HAT activity, β-catenin, and RA signalling. Intriguingly, while the association of p300 with this enhancer is less affected by RA signalling or HAT inhibition, the occupancy of β-catenin is markedly increased by RA signalling, and severely impeded by the HAT inhibition (Figure 5).

Likewise, the acetylation of H3-K27 is also induced by RA and inhibited by curcumin (Figure 5). Apparently, the HAT activity of p300 has a significant impact on β-catenin activity and H3-K27 acetylation at the *Myf5* early enhancer. Our studies shed new lights on how multiple signalling pathways converge at the *Myf5* early enhancer to potentially regulate the specification of skeletal muscle lineage. In addition, specific histone code, the acetylation of H3-K27, but not H3-K9/14 may be involved in the activation of this early enhancer. Therefore, pluripotent P19 cell differentiation provides a valuable model not only to identify small molecule inducers for directing lineage specification, but also to dissect signal-dependent activation of myogenic program on a level of transcription regulation.

In conclusion, we show here that histone acetylation and p300 activity is directly involved in the *Myf5* early enhancer at the stage of lineage specification. The intimate connection of p300 HAT activity with specific histone acetylation and β-catenin signalling at this *Myf5* enhancer during stem cell differentiation also offers new insight into the molecular basis for skeletal myogenesis in embryonic development.

## Materials and Methods

### Cell culture and differentiation

P19 stem cells (ATCC) were cultured in minimum essential medium α (Invitrogen) supplemented with 5% fetal bovine serum, 5% bovine calf serum (PAA), and Penicillin/Streptomycin (Invitrogen) at 37°C with 5% CO2. Cells were differentiated as described previously [23]. Briefly, the cells grown in Petri dishes to form EBs for four days and treated as indicated. Following EB formation, the cells were maintained in tissue culture dishes for an additional 5 days without treatments. All-*trans* retinoic acid and curcumin were purchased from Sigma-Aldrich. C646 was synthesized as described previously [26]. All reagents were dissolved in ethanol. Antibodies against p300, MyoD, Myf5, and acetylated H3K9/14 were purchased from Santa Cruz. Specific antibody for acetylated H3-K27 was from Abcam and for β-catenin was from Millipore. Hybridoma supernatants were used against myosin heavy chain (MF20), β-tubulin (E7), and myogenin (F5D). Secondary fluorescent antibodies Alexa Flor^®^488 goat antimouse, goat anti-rabbit, and Alexa Flor^®^594 donkey anti-mouse were from Invitrogen.

### Immunofluorescence microscopy

Following EB formation, the cells were grown on cover slips coated with 0.1% gelatin for 5 days without treatments, fixed with methanol at 4°C, and rehydrated in PBS at room temperature. Incubation with primary antibodies was carried out overnight at 4°C, and with fluorescent labelled secondary antibodies at room temperature for 30 minutes [40]. DNA was stained with 0.1 µg/ml of Hoechst (Molecular Probes) for 5 minutes.

Visualization and quantification were performed with Axiovert 200 M microscope, 16AxioCam HRM camera [41] and AxioVision Rel 4.6 software (Zeiss). For each coverslip, about 100 fields of view were analyzed. The efficacies of myogenic differentiation was presented as the percentages of cells positively stained for skeletal myocyte markers, such as myosin heavy chain, MyoD or Myf5, in comparison to the total cell populations [23].

### Western blot analysis

Cells were differentiated and harvested at the indicated time points, and then lysed in whole cell extract buffer containing 10 % glycerol, 50 mM Tris-HCl pH 7.6, 400 mM NaCl, 5 mM EDTA, 1 mM DTT, 1 mM PMSF, and 1 % NP-40 for 30 minutes on ice [42]. Protein concentrations were determined by a Bradford assay (Bio-Rad). Equal amounts of protein were resolved by SDS-PAGE, transferred onto Immune-Blot PVDF membrane (Bio-Rad), and then visualized by using Western Lightning™ Chemiluminescence (Perkin Elmer) reagents. Quantification of the Western blots were performed by using Scion Image software [43].

### HAT assay

Cells were treated as indicated during the EB formation and then harvested on day 4 to prepare the whole cell extracts. Endogenous p300 protein was then immunoprecipitated and evaluated for its HAT activity by using a HAT assay kit following the manufacturer’s instruction (BioVision). Sample immunoprecipitated with protein A agarose only was used as a negative control to subtract the background. Quantitative Western blot analysis of input p300 served as an internal control.

### Real-time RT-PCR

Total RNA was isolated using the Total RNA Kit I (Omega) according to the manufacturer’s protocol. Equal amounts of RNA were reverse-transcribed with High capacity cDNA Reverse Transcription kit [44]. Real-time PCR was performed by using a Power SYBR^®^ Green PCR Master mix on a 7500 Fast Real-Time PCR System (Applied Biosystems). Results were normalized to GAPDH, and analyzed by threshold cycle (Ct) comparative method [23]. The 2-ΔΔCt value was calculated, where ΔCt = Ctsample –CtGAPDH, and ΔΔC t= ΔCtsample − ΔCt reference. PCR primers:

Myf5fwd- GGCATGCCTGAATGTAACAGC;
Myf5 rev- CAATCCAAGCTGGACACGGA;
GAPDH fwd- TCGGTGTGAACGGATTTG;
GAPDH rev- GGTCTCGCTCCTGGAAGA.

### Chromatin immunoprecipitation assay

ChIP assay was performed as described previously [23,45]. Briefly, cells from the EBs were fixed with 1% of formaldehyde for 15 minutes at 37°C, quenched with 200 mM glycine and then lysed in Lysis Buffer (50 mM Tris-HCl pH 8.0, 10 mM EDTA pH 8.0, 1% SDS, 1 mM DTT, 1 mM PMSF, 5 mM sodium butyrate, and protease inhibitors) for 10 minutes on ice. The lysates were sonicated with a Bioruptor system (Diagenode) and pelleted by centrifugation at 14,000 g for 15 minutes. The supernatants were pre-cleared by incubation with DNA-blocked protein A-agarose beads (Upstate) for 1 h at 4°C, then immunoprecipitated with indicated antibodies overnight. IgG antiserum (Zymed Laboratories, Inc., CA) was used for the negative ChIP control. Undifferentiated and untreated cells were also included as negative controls. DNA was purified using Omega Bio-tek Cycle Pure Kit, PCR primers for the *Myf5* early enhancer:

EE fwd- AGAAGCGGCACACGTTGTA;
EE rev- TGGAGAAGAGTGAACATCCTTG
